# Should right ventricle dilatation during exercise have clinical implications in patients with chronic thromboembolic pulmonary hypertension? Case report

**DOI:** 10.1186/1476-7120-5-50

**Published:** 2007-12-27

**Authors:** Carlos Cotrim, Maria J Loureiro, Rita Miranda, Sofia Almeida, Ana R Almeida, Otília Simões, Pedro Cordeiro, Manuel Carrageta

**Affiliations:** 1 Cardiology Department, Garcia de Orta Hospital, Almada, Portugal; 2 Heart Failure and Pulmonary Hypertension Unit, Garcia de Orta Hospital, Almada, Portugal

## Abstract

We describe the case of a 30-year-old female patient with chronic thromboembolic pulmonary hypertension that has an excellent functional capacity under treatment with sildenafil. She did an exercise stress echocardiography that revealed marked right ventricular dilatation during exercise. This information was used for clinical decision and the authors discuss the potential utility of this echocardiographyc sign.

## Background

Dilatation of right ventricle induced by exercise was first described by Ramanath et al. [1] in a patient suspected of acute coronary syndrome that after exercise echocardiography revealed right ventricular dilatation and conducted to the correct diagnosis of acute pulmonary thromboembolism. Another patient with chronic thromboembolic pulmonary disease and severe pulmonary arterial hypertension with good response to treatment with bosentan experienced the same phenomenon [2]. We now describe a third case that, in our opinion highlights the utility of exercise echocardiography in this clinical setting.

## Case presentation

We report the case of a 30-year-old female patient, with a previous history of chronic pulmonary thromboembolism (PTE) and pulmonary arterial hypertension. She had C and S Protein deficit diagnosed at the first episode of PTE two years ago. She was referred to our Unit for study and treatment.

The distance attained on the six-minute walk test at the first appointment was 516 meters.

**Figure 1:**
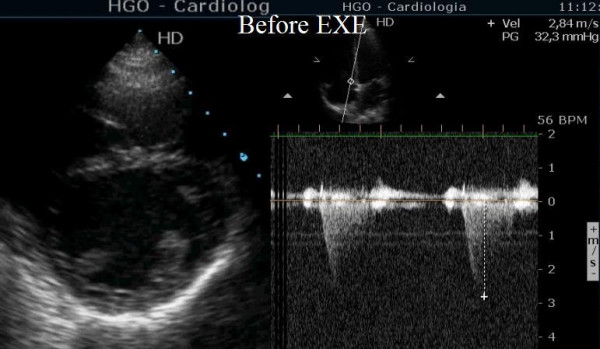
Short axis view before exercise showing normal eccentricity index of left ventricle and normal pulmonary artery pressure.

Following right heart catheterization that revealed right atrial pressure of 9 mmHg, mean pulmonary arterial pressure of 31 mmHg, cardiac output of 2,6 l/min, pulmonary wedge pressure of 8 mmHg and pulmonary vascular resistance of 704 dynes.s.cm^-5 ^we verified that there was absence of vasodilation to adenosine infusion. She started therapy with bosentan 62,5 mg twice daily. On third day of therapy the hepatic enzymes increased by more than ten times the normal value and bosentan was replaced by sildenafil 25 mg three times daily. This produced a good therapeutic response with clinical improvement, as shown by a 600 meter with the six-minute walk test and a gradient between right ventricle and right atrium of 32 mmHg and a normal eccentricity index of left ventricle (Fig. 1 and Additional file 1).

**Figure 2:**
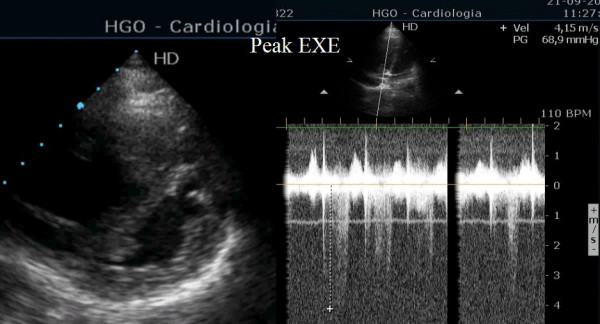
Short axis view at peak exercise showing alteration of eccentricity index of left ventricle, dilatation of right ventricle and severe pulmonary hypertension.

**Figure 3:**
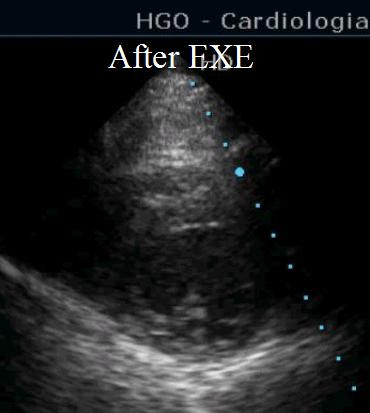
Short axis view shortly after exercise with normal dimensions and morphology of left and right ventricle.

We performed Doppler exercise echocardiography [3] using the modified Bruce protocol. Right ventricular dilatation was observed near the peak exercise with an increased pulmonary artery systolic pressure (from 32 to 69 mmHg + right atrial pressure) and an eccentricity index profoundly alterated (Fig. 2 and Additional file 2). The short axis view obtained 40 seconds after exercise was normal again (Fig. 3 and Additional file 3).

The marked changes observed during exercise echocardiography conducted to pulmonary angiography that showed significant alterations in the main branches of the pulmonary artery. The patient was referred to pulmonary endarterectomy.

## Discussion and conclusion

Treatment of pulmonary arterial hypertension secondary to chronic pulmonary thromboembolic disease is challenging and according to the established guidelines [4] for pulmonary thromboendarterectomy should be considered for patients with the following criteria: 1) NYHA functional class III or IV symptoms; 2) a preoperative pulmonary vascular resistance greater than 300 dynes.s.cm^-5^, or 800 dynes.s.cm^-5 ^according to other authors [5]; 3) surgical accessible thrombus (in main, lobar, or segmental pulmonary arteries), as determined by the appropriate studies; and 4) no severe comorbidities. In addition, some centres have suggested that mean PAP preoperatively should be at least ≥ 40 mmHg.

Our patient clearly doesn't fill the first criteria since under treatment [6] with sildenafil 25 mg three times daily she has an excellent 6 MWT of 600 meters. However she is young and complains of exertional dyspnoea. Besides exercise echocardiography disclose seriously impaired pulmonary vascular vasodilator reserve. We admit that, despite clinical benignity, the patient still had severely limited pulmonary vasodilator reserve and we performed pulmonary angiography.

In face of the result of the exercise echo we decided to refer the patient to a centre with experience in pulmonary endarterectomy.

The presence or absence of this sign – right ventricular dilatation with exercise – should, in our opinion, be evaluated in patients with chronic pulmonary thromboembolic disease and pulmonary hypertension whenever there are uncertainties about the indication for surgical pulmonary endarterectomy. We think that this sign is probably ominous and should almost certainly reinforce the indication for surgery.

The acute morphologic changes in cardiac anatomy reflect the changes in right and left ventricular haemodynamics,. Echocardiography might be helpful in evaluating the acute hemodynamic response to pulmonary output increase that exercise elicits. The practical utility of this concept for clinical decision is demonstrated in this case report

## Authors' contributions

CC performed exercise echocardiography, review literature and wrote the manuscript.

MJL, RM, SA, OS, PC are responsible for clinical assessment of the patient, participate in drafting, and revised the manuscript for important intellectual content.

ARA revised the manuscript for important intellectual content.

MC gave final approval to the manuscript.

All authors read and approved the final manuscript.

## Supplementary Material

Additional file 1 Short axis view before exercise. Short axis view showing almost normal morphology of left and right ventricles Click here for file

Additional file 2 Short axis view at peak exercise. Short axis view showing marked dilatation of right ventricle and huge alteration of eccentricity of left ventricle. Click here for file

Additional file 3 Short axis view shortly after exercise. Short axis view shortly after exercise showing normalization of cardiac morphology Click here for file
